# White and non-White Australian mental health care practitioners’ desirable responding, cultural competence, and racial/ethnic attitudes

**DOI:** 10.1186/s40359-022-00818-4

**Published:** 2022-05-07

**Authors:** Tinashe Dune, Ritesh Chimoriya, Peter Caputi, Catherine MacPhail, Katarzyna Olcon, Anita Ogbeide

**Affiliations:** 1 School of Health Sciences, Western Sydney University, Campbelltown, NSW 2560 Australia; 2 Translational Health Research Institute, Western Sydney University, Campbelltown, NSW 2560 Australia; 3 Diabetes, Obesity and Metabolism Translational Research Unit, Western Sydney University, Campbelltown, NSW 2560 Australia; 4 School of Medicine, Western Sydney University, Campbelltown, NSW 2560 Australia; 5 School of Psychology, University of Wollongong, Wollongong, NSW 2522 Australia; 6 School of Health and Society, University of Wollongong, Wollongong, NSW 2522 Australia

**Keywords:** Mental health, Practitioner, Desirable responding, Cultural competence, Whiteness, Australia, Racial and ethnic blindness

## Abstract

**Background:**

Racial, ethnic, religious, and cultural diversity in Australia is rapidly increasing. Although Indigenous Australians account for only approximately 3.5% of the country’s population, over 50% of Australians were born overseas or have at least one migrant parent. Migration accounts for over 60% of Australia’s population growth, with migration from Asia, Sub-Saharan African and the Americas increasing by 500% in the last decade. Little is known about Australian mental health care practitioners’ attitudes toward this diversity and their level of cultural competence.

**Aim:**

Given the relationship between practitioner cultural competence and the mental health outcomes of non-White clients, this study aimed to identify factors that influence non-White and White practitioners’ cultural competence.

**Methods:**

An online questionnaire was completed by 139 Australian mental health practitioners. The measures included: the Balanced Inventory of Desirable Responding (BIDR); the Multicultural Counselling Inventory (MCI); and the Color-blind Racial Attitudes Scale (CoBRAS). Descriptive statistics were used to summarise participants’ demographic characteristics. One-way ANOVA and Kruskal–Wallis tests were conducted to identify between-group differences (non-White compared to White practitioners) in cultural competence and racial and ethnic blindness. Correlation analyses were conducted to determine the association between participants’ gender or age and cultural competence. Hierarchical multiple regression analysis was conducted to predict cultural competence.

**Results:**

The study demonstrates that non-White mental health practitioners are more culturally aware and have better multicultural counselling relationships with non-White people than their White counterparts. Higher MCI total scores (measuring cultural competence) were associated with older age, greater attendance of cultural competence-related trainings and increased awareness of general and pervasive racial and/or ethnic discrimination. Practitioners with higher MCI total scores were also likely to think more highly of themselves (e.g., have higher self-deceptive positive enhancement scores on the BIDR) than those with lower MCI total scores.

**Conclusion:**

The findings highlight that the current one-size-fits-all and skills-development approach to cultural competence training ignores the significant role that practitioner diversity and differences play. The recommendations from this study can inform clinical educators and supervisors about the importance of continuing professional development relevant to practitioners’ age, racial/ethnic background and practitioner engagement with prior cultural competence training.

## Background

Australia is increasingly known for its diversity and multiculturalism. By June 2020, there were over 7.6 million migrants living in Australia, 29.8% of Australia's population were born overseas and Australia's population increased by 194,400 people due to net overseas migration [1]. This is in addition to Australia’s 798,400 Aboriginal and Torres Strait Islander people, which was a 19% increase between 2011 and 2016 [2]. While the region of largest immigration remains the United Kingdom, migration from Asia, Sub-Saharan Africa and the Americas has increased by 500% in the last 10 years [2]. Sixty percent of migrants, who can demonstrate high levels of English proficiency, educational attainment, and financial independence, arrive to Australia on skilled worker and/or study visas with an additional 30% arriving as partners or family to these migrants. Australia also has a humanitarian visa program which accounts for less than 10% of Australian migration and includes asylum seekers who may have limited English, education, and access to financial support [2]. This level of diversity requires the Australian society as well as its systems, services, and support providers to consistently adapt to meet the needs of a rapidly changing population. However, despite increased diversity, Australian society and its health care systems continue to operate within the framework of Whiteness^1^ [4, 5].

### Whiteness in Western society and mental health care

Indigenous scholars like Moreton-Robinson [6–9] demonstrate the insidious role and persistence of Whiteness since the invasion of Australia by the British in 1788 through to the present day. Scholars focused on the study of Whiteness assert that it is a colonial and post-colonial ideology and a power structure characterised by racial dichotomies and ethnic hierarchies [10–13]. DiAngelo [14], for example, explains that “Whiteness itself refers to the specific dimensions of racism that serve to elevate White people over people of color” (p. 56). As such, other scholars indicate that Whiteness leads Whites to believe themselves to be the “predestined master[s] of the world” [15]. These definitions of Whiteness clarify that across historical and contemporary Australian society, those who reap the benefits of Whiteness are those who are White [8, 9]. Hence, in this paper, the term non-White^2^ refers to individuals who are excluded from being beneficiaries of Whiteness as a result of their racial, ethnic, cultural, religious, linguistic, or national identities [13, 16, 17]. This definition of “non-White” also includes individuals who may appear White yet identify as being of an ethnic minority because they experience prejudice or discrimination due to other aspects of their identity (e.g., wearing a hijab, having a non-Australian accent, or having non-Anglo family members).

We recognise that not all Whites encounter the same privileges, or that Whiteness has maintained the same parameters over time, or that it is impermeable and inflexible [18, 19]. Additionally, we recognise that Whiteness (and how those who benefit, or are excluded, from it are defined) is constantly under revision [20]. Even within this consistent state of flux, Whiteness and its consequences endure [21] and are present across all areas of Western (if not global) environments, systems, and practices [22].

One such system is the mental health care system. Mental health is a core element of human health and wellbeing. In 2020, the Australian Institute of Health and Welfare (AIHW) [23] indicated that 45% of all Australians aged 16 to 85 years—8.7 million people—will experience mental illness at some point in their life. According to 2015 statistics, mental illness and substance misuse were the second largest contributor (23%) of the non-fatal burden of disease in Australia [24]. During the 2017 and 2018 financial year, $9.9 billion [23] was spent on mental health, with spending increasing in 2018–2019 to $10.6 billion [25]. Given the prevalence and social and economic costs of mental illness, access to and effectiveness of mental health services is crucial. Mental health is therefore an area in which systems and practitioners need to adapt and demonstrate flexibility to support clients [26].

#### The impact of Whiteness on mental health systems and outcomes

Despite consistent efforts and improvements, (mental) health practitioners and systems struggle to adapt or to be flexible when working with clients who do not fit within rigid frameworks of Whiteness in health care [4, 5]. The rigidity of Whiteness in health care is evident, for example, in the medicalisation and pathologising of social experiences (like racism and discrimination) into mental health problems (e.g., schizophrenia) which are then relegated to the individual to overcome through (largely) self-funded talk-therapy and medication [27]. For instance, a 2019 U.S. study of 599 Blacks and 1058 non-Latino Whites, found that mental health clinicians failed to effectively consider mood symptoms when diagnosing schizophrenia among African-Americans [28]. The study authors indicated that the findings suggest the presence of racial bias, whether conscious or subconscious, as a factor in the comparatively higher levels of diagnosis of schizophrenia in Black populations. Despite the fact that discrimination based on race, ethnicity, culture or religion is a key contributing factor in the onset of and severity of disease—resulting in disparities in physical and mental health among non-White people—mental health practitioners continue to manifest prejudicial attitudes that hinder equitable health care and improved health outcomes for non-White people [29].

#### From overt to covert racism and discrimination: colour-blind attitudes and beliefs

The negative health outcomes and experiences of non-Whites within (mental) health care settings are well documented and relate to systems and practitioners that harbor prejudicial attitudes and beliefs about non-White people. While contemporary attitudes and beliefs about non-White people are moving away from overtly racist (for the most part) manifestations of discrimination, covert forms of racism persist [5]. A notable example of covert racism are colour-blind attitudes and beliefs [20, 30]. These include the assertion that everyone is equal, racism is no longer a problem and that all people (regardless of their race, ethnicity, religion, language or culture) can achieve the same social, political, economic and health outcomes [20]. However, this widely researched perspective has been determined to be a manifestation of Whiteness whereby race, privilege and power are erased to avoid the discomfort of explicitly naming and identifying responsibility for addressing Whiteness [20, 30–33].

Evidence demonstrates that mental health practitioners that espouse colour-blind racial and/or ethnic attitudes have lower levels of cultural competence [34–37]. This was a key finding in our 2018 systematic review, representing 5870 mental health care practitioners, that explored practitioner attitudes and beliefs about non-White people. The review found that practitioners who perceived non-White people negatively or through a colour-blind belief system were less likely to engage in culturally competent health care approaches while working with non-White people [34]. However, most of the studies in the aforementioned systematic review were from the United States (twenty-eight), and only three were from Australia. Given Australia’s increasing diversity, it is important to consider and identify Australian mental health practitioners’ attitudes and beliefs about non-White people and their impact on cultural competence and the counselling relationship. In doing so, advances can be made in Australian (and international) practitioner training and clients’ mental health outcomes. However, consideration about how such an exploration is conducted is important to ensure that the data collected from mental health practitioners’ self-reports are not confounded by socially desirable responding [32].

#### Whiteness and socially desirable responding

Given that this study is interested in understanding practitioner perspectives about non-White people, some practitioners may be inclined to respond in socially desirable ways in order to downplay or underrepresent their true attitudes and beliefs [32]. Desirable responding in relation to perceptions of non-White people therefore helps an individual distance themselves from the guilt, frustration and anxiety about as well as their role and responsibility in the perpetuation of Whiteness [32, 33, 38–40]. As such, evidence indicates that assessing for social desirability is integral to interpreting cultural competence self-report data [41]. To assess and moderate for this possibility, research exploring practitioner self-report cultural competence data often includes specific questionnaires that measure participants’ propensity to respond in desirable ways. In this study, desirable responding was assessed in line with international research indicating that people are more likely to respond in deceptive and/or desirable ways due to the sense of confrontation and discomfort that often results from thinking or talking about Whiteness, racism, privilege and their impacts on non-White people [32, 33, 38–40].

### Solutions to the impact of Whiteness in mental health care

To manage and reduce the impact of Whiteness and its perpetuation in mental health care, practitioners and researchers advocate for open discourse about Whiteness, racism, privilege, supremacy and power imbalances via cultural competence training [22]. Cultural competency is defined as “a set of congruent behaviours, attitudes and policies that come together in a system, agency or among professionals and enable that system, agency or those professionals to work effectively in cross-cultural situations” [43].^3^ When taught comprehensively (e.g., inclusive of in-depth discussions about Whiteness, its impacts and the practitioner’s role in its manifestations and management), the skills, knowledge and awareness gained within cultural competency training helps to reduce barriers to trust and power imbalances between clients and practitioners thereby improving the therapeutic relationship [44]. In a 2019 U.S. Delphi study exploring mental health care providers training needs, Baima and Sude [11] found that the panel of 20 practitioners of diverse ethnic backgrounds endorsed the need for mental health professionals to understand historical and contemporary Whiteness within the mental health fields and larger social systems. The panel of participants also indicated the need for the therapist to understand their role in Whiteness as well as the need for practitioners to identify the challenges to understanding Whiteness within clinical training and practice. Importantly, the panel of practitioners in that study identified that mental health practitioners needed to transform their cognitive and emotional attitudes and beliefs in order to address Whiteness within the context of mental health service provision and to support client trust and participation in therapy. These findings align with international evidence indicating that when practitioners internalise and enact the principles of cultural competence, they are able to cultivate and maintain a positive multi-cultural counselling relationship between themselves and their clients [22, 45–47].

**Fig. 1:**
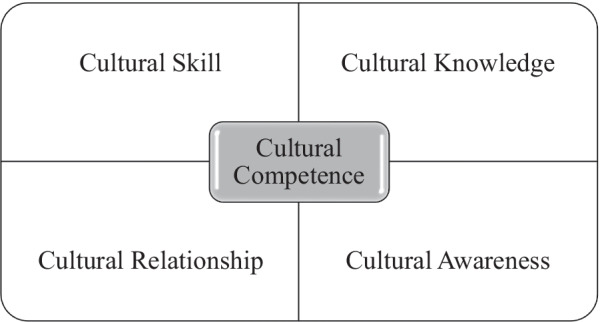
Operationalising cultural competence

The counselling relationship is broadly defined as “the collaborative and affective bond between therapist and patient … [and] is an essential element of the therapeutic process” [48]. This relationship is exemplified by bi-directional and mutual collaborations between mental health care providers and clients, are dependent on a person-centred health care ethos [49] and based on a mutually nurtured psycho-socio-emotional connection [50]. While the concept of the counselling relationship has its origins in early psychoanalytic theories (e.g., [51]), it is now a staple of contemporary evaluations and conceptualisations of the therapeutic process. Notably, international evidence indicates that the multi-cultural counselling relationship is a core component of cultural competence and is strongly associated with clients’ mental health engagement and outcomes [48, 52]. Operationally then, culturally competent mental health care is inclusive of an effective counselling relationship and requires practitioners to consistently utilise and improve on their cross- and multi-cultural skills, knowledge, awareness and relationships with non-White people [53]. Figure 1 illustrates these components of cultural competence.

Importantly, international research demonstrates that practitioner cultural competence is dependent on a range of factors. The most notable factors include mental health practitioner race/ethnicity and engagement in cultural competence training. For instance, a U.S. study of 220 counsellors found that Asian and Latino counsellors reported having higher multi-cultural knowledge than White counsellors and Black, Asian and Latino counsellors reported more competence in multi-cultural awareness and relationships than did White counsellors [54]. However, comprehensive cultural competence training can moderate this discrepancy in cultural competency between White and non-White practitioners [32].

### Significance of the current study and the Australian context

In Australia, little is known regarding mental health workers’ attitudes towards non-White people let alone the factors that influence practitioner cultural competence. While health care provider attitudes can be implied from the well documented negative health experiences and outcomes of non-White populations in Australia [55], a better understanding of Australian mental health care practitioners' attitudes and beliefs about non-White people and their cultural competence is needed. By identifying and exploring this gap, robust recommendations for practitioner training, practice and research can be developed with the aim of improving non-White people’s mental healthcare experiences and outcomes.

Given that this study is the first of its kind in Australia, we have followed the structure of international research from other Western nations which compare and contrast desirable responding, cultural competence, and colour-blind racial and ethnic attitudes across White and non-White practitioners [30, 33, 54, 56, 57]. Collecting and comparing racial/ethnic data in line with international research is important because Australia does not currently collect robust data about practitioner ethnicity. This failure to collect basic demographics about Australian mental health care practitioners attests to the impact of Whiteness, which erases difference within the health care system and workforce [13, 20, 27, 58]. Given the limited data available in Australia, the following example of Australian registered psychologists is used to illustrate the dearth of demographic information.

According to Mathews, Stokes, Crea, and Grenyer [59], of the 11,897 psychologists who completed the 2008 Australian Psychology Workforce Survey, only 54 were of Indigenous background. The authors also found that minority population groups such as culturally and linguistically diverse and Indigenous clients made up 54.65% and 15.99%, respectively, of psychologists’ workloads—a significant proportion. It can therefore be assumed that with Australian migration having increased significantly within the last decade, the number of non-White clients has also increased. More recently, the Department of Health [60] reported that out of 26,311 registered psychologists, 73.3% were born in Australia and 0.7% were of Aboriginal and Torres Strait Islander background. There is no clear data on the ethnicity of psychologists beyond Indigeneity in Australia—giving the impression that, besides being Indigenous, the remainder of psychologists are White Australians. However, drawing on data from the Australian Bureau of Statistics [61], it is likely that at least 20% of the Australian born psychologists are likely to have at least one parent who was born overseas. Psychology has, however, typically been a profession dominated by people from White backgrounds [62, 63]. As such, the number of non-White psychologists is not likely to be representative of the actual diversity in Australia’s population [63, 64]. This omission of diversity in the collection of demographic data regarding the Australian mental health workforce is an example of how frameworks of Whiteness perpetuate racial hierarchies and the erasure of difference within Australian society.

This study therefore focused on the following research questions comparing White and non-White practitioners:

1. To what degree do Australian mental health care practitioners respond in socially desirable ways?
2. How culturally competent are Australian mental health care practitioners?
3. To what degree do Australian mental health practitioners ascribe to colour-blind racial and ethnic attitudes?
4. What variables (e.g., desirable responding, demographics, experience with non-White clients, attendance at cultural competence training, and racial/ethnic attitudes) predict cultural competence?

## Methods

This quantitative study is part of a larger body of work using a sequential mixed methods design (see publications [18, 19, 34]). The current study used a cross-sectional design with non-probability sampling and validated survey measures.

### Recruitment and participants

Participants included individuals aged 18 years and over, living in Australia and self-identifying as a mental health care practitioner or trainee. Participants were recruited via distribution of advertising to the Australian Psychological Society and the Australian Clinical Psychologists Association. Additional recruitment took place via social media including Facebook, Twitter, Instagram, and LinkedIn. Participants were invited to complete the survey online or request a paper copy. The purpose of the study was described to the participants as an exploration of practitioner cultural competence and attitudes towards non-White clients.

One hundred and eighty-two practitioners commenced the survey. The majority engaged in the survey online (n = 175) while seven elected to complete the survey in hardcopy. After screening for completeness of all the demographic, MCI, CoBRAS and BIDR items, 139 participants remained. The study was approved by and conducted within the guidelines of the University’s Human Research Ethics Committee (Approval Number 2017/105) at the University of Wollongong.

### Quantitative survey measures

The survey included the following measures:

#### Demographic questionnaire

The demographic questions collected information about participants’ age, gender, ethnicity and country of origin, highest degree achieved, year highest degree achieved, mode of professional practice/training (private and/or public), years of experience and engagement with post-qualification cultural competency training. Participants were also asked where they completed or are engaged in their mental health training and how many non-White clients they see in a month.

#### Balanced Inventory of Desirable Responding

The Balanced Inventory of Desirable Responding (BIDR) [65] measures the tendency to respond and exhibit behaviours or thoughts that are viewed as socially desirable but are not accurate representations of an individual’s real attitudes and behaviours [66]. Scholars have used BIDR to assess and control for counsellors’ social desirability in self-report data [33].

The BIDR consists of 40 items and two subscales, each with 20 items. Subscale 1: Self Deceptive Enhancement (SDE) measures positive self-descriptions that a respondent actually believes to be true [67]. The SDE subscale consists of the first 20 items of the BIDR. Example SDE items include: “My first impressions of people usually turn out to be right”, “I never regret my decisions” and “I always know why I like things.” Subscale 2: Impression Management (IM) measures a respondent’s conscious biasing of responses to create a favourable self-image [67]. The IM subscale consists of the last 20 items of the BIDR. Example IM items include: “I never swear”, “I always declare everything at customs” and “I never take things that don’t belong to me.”

The BIDR is composed of a response format consisting of a 7-point Likert scale ranging from 1 (not true) to 7 (very true); 1 point is scored for each extreme answer (6 or 7) with a total score ranging from 0 to 40. Higher scores indicate a greater tendency to respond and exhibit behaviours or thoughts that are viewed as socially desirable. The BIDR has been used successfully with various racial/ethnic and cultural groups [33]. Chao [32] reported a coefficient alpha of 0.85 with a sample of graduate students in psychology and mental health professions. Our study reports the coefficient alpha of 0.88 for total score, 0.73 for Subscale 1: Self Deceptive Enhancement, and 0.86 for Subscale 2: Impression Management. This questionnaire is a valid measure and has been used extensively within health and education professionals internationally [68–70].

#### Multicultural Counselling Inventory (MCI)

This 40-item self-report inventory [71] assesses behaviours and attitudes related to four multicultural competencies on a 4-point Likert scale from very inaccurate (1) to very accurate (4). For each item, a score of 1 indicates low multicultural competence and a score of 4 indicates high multicultural competence [41]. Scale scores are obtained by adding the items specific to each subscale. The MCI total score ranges from 40 to 160 with higher scores indicating greater multicultural competence. Higher subscale scores also indicate greater multicultural competence in the respective subscale areas (see below). In this study, the MCI total score is the dependant variable. As explained in Ottavi et al. [72], the four areas of multicultural competency are as follows:

1. Skills—11 items measuring general counselling and specific multicultural counselling skills. Sample items include “When working with all clients, I am able to be concise and to the point when reflecting, clarifying, and probing” and “When working with minority clients, I monitor and correct my defensiveness.” Minimum score = 11, maximum score = 44.
2. Knowledge—11 items measuring treatment planning, case conceptualisation, and multicultural counselling research. Sample items include “When working with minority clients, I keep in mind research findings about minority clients’ preferences in counselling” and “When working with minority clients, I apply the socio-political history of the clients’ respective minority groups to understand them better.” Minimum score = 11, maximum score = 44.
3. Awareness—10 items measuring multicultural sensitivity, interactions, and advocacy in general life experiences and professional activities. Sample items include “I am involved in advocacy efforts against institutional barriers in mental health services for minority clients” and “When working with international students or immigrants, I understand the importance of legalities of visa, passport, green card and naturalization.” Minimum score = 10, maximum score = 40.
4. Relationship—8 items measuring the counsellor's interaction process with the minority client (e.g., comfort level, worldview, and counsellor’s trustworthiness). Sample items include “When working with minority individuals, I am confident that my conceptualization of individual problems do not consist of stereotypes and biases” and “When working with minority clients, I perceive that my race causes the client to mistrust me.” Minimum score = 8, maximum score = 32.

Internal consistency reliabilities (Cronbach’s alphas) reported by Sodowsky et al. [71] were 0.80 for Multicultural Awareness, 0.80 for Multicultural Counselling Knowledge, 0.81 for Multicultural Counselling Skills, 0.67 for Multicultural Counselling Relationship, and 0.86 for the full scale. Our study reports 0.78 for Multicultural Awareness, 0.83 for Multicultural Counselling Knowledge, 0.87 for Multicultural Counselling Skills, 0.69 for Multicultural Counselling Relationship, and 0.90 for the full scale. This measure is valid and has been used extensively with diverse groups of counsellors and therapists [73–75].

#### Color-Blind Racial Attitudes Scale (CoBRAS)

This study used an adapted version of the Color-Blind Racial Attitudes Scale [31] to assess practitioners’ constructions of non-White people. The CoBRAS consists of 20 items to assess attitudes with a 6-point Likert scale of 1 (strongly disagree) to 6 (strongly agree). The CoBRAS was designed to assess cognitive dimensions of colour-blind racial attitudes including the degree to which respondents distort, deny, and/or minimise the existence of institutional racism. Sample items include: “Everyone who works hard, no matter what race they are, has an equal chance to become rich,” and “Racism may have been a problem in the past, but it is not an important problem today”. Total scores can range from 20 to 120, with higher scores representing greater colour-blind racial beliefs [31] which are an indication of greater levels of unawareness or blindness to the existence and impact of Whiteness on people and systems.

The CoBRAS assesses blindness in three areas: Racial & Ethnic Privileges, Institutional Discrimination, and Blatant Racial & Ethnic Issues. The Racial & Ethnic Privilege subscale measures blindness to the existence of privileges attributed to Anglo-Australians. The Institutional Discrimination subscale measures limited awareness of the implications of institutional discrimination and exclusion. The Blatant Racial and Ethnic Issues subscale measures unawareness of general and pervasive racial/ethnic discrimination.

Although the CoBRAS is based on the U.S. context and uses terminology relevant to racial dynamics in the U.S., many of these dynamics (e.g., institutional racism and systemic discrimination) are also present within the Australian context. As such, the content of the items did not require amendment, however their context ‘American’ versus ‘Australian’ or ‘United States’ versus ‘Australia’ or ‘African American’ versus ‘Afro-Australian’ did require revision. In place of the term ‘White’, the term ‘Anglo-Australian’ was used as many individuals who identify as being of an ethnic minority may appear White but may experience prejudice or discrimination due to other manifestations of their ethnicity (wearing a hijab, having a non-Australian accent, or having non-Anglo family members for example). Finally, for those items which only mention race, and not race and ethnicity, the word ethnicity or ethnic has been added to reflect the realities of multiculturalism in Australia where ethnicity may be mutually exclusive of race (e.g., “Racial problems in the U.S. are rare, isolated situations” was changed to “Racial problems in Australia are rare, isolated situations”. American spellings (e.g., color versus colour) were also amended in line with Australian spelling conventions.

Neville et al. [31] reported that the coefficient alpha for the total CoBRAS was 0.91. Our study demonstrates the coefficient alpha for the total CoBRAS was 0.87, Factor: Unawareness of Racial Privilege was 0.78, Factor: Unawareness of Institutional Discrimination was 0.71, and Factor: Unawareness of Blatant Racism Issues was 0.71. This questionnaire is a valid measure and has been used extensively with diverse U.S helping professionals and within lay populations [30–33, 76, 77].

### Data analysis strategy

Sample size was estimated to be *N* = 134 (at 90% power, α = 0.05, β = 0.1, anticipated MCI mean total score in study population = 122), which is adequate for anticipated effect size on the basis of previously published literature [78, 79]. A sample of *N* = 139 was recruited in this study, which also appears to be appropriate according to Cohen’s guideline which suggests *N* = 97 is adequate for medium effect size [80]. Reliability analyses were also conducted to assess internal consistency of each measure and to ensure their reliability within this novel sample. Descriptive statistics were used to summarise participants’ demographic characteristics. Kolmogorov–Smirnov test and Shapiro–Wilk test were conducted to check the normality of the data. One-way ANOVA and Kruskal–Wallis test were performed to identify the between-group (White and non-White practitioners) differences in socially desirable responding, cultural competence and racial and ethnic blindness (research questions 1, 2, and 3). A correlation analysis was conducted to determine the association (if any) between participants’ gender, age and cultural competence (MCI total score) (research question 4).

Following steps similar to those used by Chao [32, 33], who conducted a similar analysis amongst school counsellors in the U.S., we performed a hierarchical multiple regression analysis to predict practitioner cultural competence (MCI total score). Furthermore, a hierarchical regression analysis allowed to observe whether adding a variable significantly improves a model’s ability to predict criterion variable. A linear relation between the independent variable and dependent variables were observed prior to the consideration to the model. In the first step, social desirability (measured by BIDR subscales) was entered to elucidate the relation of social desirability with the cultural competence. In the second step, participants’ age, ethnicity (for the two groups—White and non-White^4^—entered with dummy codes), and participants’ speaking a language other than English were entered. This step demonstrated the impact of demographic variable on the relation between social desirability with the cultural competence. In the third step, cultural competency variables including the number of cultural competence-related workshops, conferences or training sessions attended since beginning practicing mental health care, number of non-White clients^5^ seen a week, and whether their formal training prepared them to work with non-White clients were entered. Anticipating the impact of the cultural competency variable on the existing model, step 3 was adopted to demonstrate the impact of those variable in the criterion variable. In the fourth step, racial and ethnic blindness attitudes (measured by CoBRAS subscales) were entered to demonstrate the impact of color-blind racial attitude in the model. This analysis addressed research question 4.

## Results

Table 1 summarises the demographic, professional, and cultural competency related characteristics of the participants. The mean age of the participants was 37.3 (*SD* = 11.3) years, with majority being female (89.2%). Most of the participants were White (64.7%), and an Australian citizen (91.4%). Most participants had completed a postgraduate degree (80.6%) and had completed their highest level of academic qualification within last five years (59.7%). All participants received their academic qualifications from Commonwealth countries, and the majority did not speak a language other than English (66.2%). Of the 139 participants, 76.7% were psychologists with varied duration working in mental health and types of practice. In terms of cultural competency related characteristics, most participants (93.5%) were seeing at least one non-White client per week, 82% of the participants had attended cultural competence-related workshops, conferences, or training sessions since the beginning of their mental health care practice, and over half of the participants (51.5%) agreed that their qualifying mental health training sufficiently prepared them to provide culturally competent services for non-White clients.

**Table 1:** Practitioners’ demographic, professional, and cultural competency related characteristics (*n* = 139)

Variable	n (%) or mean (SD)
Demographic characteristics	
Age (in years)	37.3 (11.3)
Gender	
Female	124 (89.2%)
Male	14 (10.1%)
Other	1 (0.7%)
Ethnicity	
Asian	19 (13.7%)
African	4 (2.9%)
Latin American	6 (4.3%)
Eastern European	13 (9.4%)
Aboriginal and/or Torres Strait islander	5 (3.6%)
Middle Eastern	4 (2.9%)
Mediterranean	12 (8.6%)
White	90 (64.7%)
Australian Residency Status	
Australian citizen	127 (91.4%)
Australian permanent resident	3 (2.2%)
Work visa holder	3 (2.2%)
Student visa holder	4 (2.9%)
Other (temporary visa holder and New Zealand citizen)	2 (1.4%)
Professional (cultural competency and other work related) characteristics	
Highest level of education	
Completed postgraduate degree	112 (80.6%)
Completed university degree	25 (18.0%)
Not completed degree at university	1 (0.7%)
Completed high school diploma or equivalent	1 (0.7%)
Year of obtaining qualification	
Within last 5 years (since 2016)	83 (59.7%)
Before 5 years	54 (38.8%)
Participants speaks another language in addition to English	
Yes	47 (33.8%)
No	92 (66.2%)
Occupational role	
Clinical psychologist	58 (41.7%)
Psychologist (general)	36 (25.6%)
Psychology trainee (provisional)	13 (9.4%)
Social worker (or trainee)	6 (4.3%)
Counsellor (or trainee)	7 (2.9%)
Other (Youth/ Community worker, Health Promotion Officer, Medical student, Psychotherapist/or trainee, Pharmacist, Allied health managers, Occupational therapist, Clinical psychologist registrar, Nurse practitioner and Psychology student)	19 (13.7%)
Years working in mental health	
0–1 years	19 (13.7%)
2–5 years	52 (37.4%)
6–10 years	27 (19.4%)
11–14 years	12 (8.6%)
≥ 15 years	28 (20.1%)
Type of service	
Private practice	59 (42.4%)
Public practice	48 (34.5%)
Both private and public practice	21 (15.1%)
Other (none, NGO, disability services, project consulting, research, community clinic)	5 (3.6%)
Both private and other practice	3 (2.2%)
Cultural competency experience variable	
Number of non-White clients per week	
None	9 (6.5%)
1–3	59 (42.4%)
4–6	39 (28.1%)
7–9	16 (11.5%)
≥ 10	16 (11.5%)
Engagement in cultural competence-related workshops, conferences or training since beginning practicing mental health care	
None	25 (18.0%)
1–3	75 (54.0%)
4–6	30 (21.6%)
7–9	3 (2.2%)
≥ 10	6 (4.3%)
Did your professional mental health training sufficiently prepare you to provide culturally competent services for non-White clients	
Agree	71 (51.1%)
Neutral	18 (12.9%)
Disagree	50 (36.0%)

To examine whether the cultural competence as measured by MCI total score varied as a function of participants’ gender and age, we conducted an analysis of variance (ANOVA) of gender and a correlation analysis of age with the dependent variables. The ANOVA results revealed no significant main effects for participants’ gender on MCI total score, *F*(2,139) = 0.863, *p* = 0.424. A correlation analysis showed that age was weakly but statistically significantly correlated with the total score of MCI (*r* = 0.183, *p* = 0.032) and thus was considered a predictor variable in the final analysis.

### Differences in desirable responding across White and non-White practitioners

The average total BIDR score of all participants was *M* = 10.4 (*SD* = 6.8) (Table 2). The total score was not statistically different across the two groups despite the non-White group having a higher score than the White group (*M* = 11.8, *SD* = 7.2 vs. *M* = 9.6, *SD* = 6.3, *p* = 0.094). This finding suggests that the groups did not differ in terms of desirable responding. No significant multicollinearity was observed in the model (Variance Inflation Factor -VIF for BIDR subscale 1 = 1.44 and BIDR subscale 2 = 1.41).

**Table 2:** Differences between White and non-White practitioners’ cultural competence, desirable responding, and racial and ethnic blindness

Scores/variable Mean (SD)	All participants (*n* = 139)	White (*n* = 90)	Non-White (*n* = 49)	*p* value
Age	37.3 (11.3)	38.5 (11.6)	34.7 (10.2)	0.077*
Multicultural Counselling Inventory (MCI)				
Composite score (MCI total)	122.9 (14.0)	121.5 (13.6)	125.5 (14.6)	0.069
Subscale 1: Multicultural Counselling Skills	37.1 (5.0)	37.3 (5.0)	36.7 (5.0)	0.554
Subscale 2: Multicultural Awareness	27.5 (5.0)	26.4 (4.6)	29.5 (5.0)	< 0.001*
Subscale 3: Multicultural Counselling Relationship	27.5(5.0)	24.2 (3.3)	25.4 (3.5)	0.049*
Subscale 4: Multicultural Counselling Knowledge	33.7 (4.8)	33.7 (4.5)	33.9 (5.4)	0.433
Balanced Inventory of Desirable Responding (BIDR)				
Composite score (BIDR Total)	10.4 (6.8)	9.6 (6.3)	11.8 (7.2)	0.094
Subscale 1: Self Deceptive Enhancement (SDE)	4.2 (3.1)	3.9 (2.9)	4.8 (3.2)	0.135
Subscale 2: Impression Management (IM)	6.2 (4.5)	5.7 (4.3)	7.0 (4.8)	0.132
Color-Blind Racial Attitudes Scale (CoBRAS)				
Composite score (CoBRAS Total)	43.8 (13.6)	41.5 (13.6)	48.0 (13.0)	0.007*
Factor 1 (URP-Unawareness of Racial Privilege)	18.8 (6.4)	17.6 (6.2)	20.9 (6.4)	0.003*
Factor 2 (UID-Unawareness of Institutional Discrimination)	14.4 (5.3)	13.9 (5.5)	15.4 (4.9)	0.059
Factor 3 (UBRI-Unawareness of Blatant Racism Issues)	10.6 (4.3)	10.0 (4.0)	11.9 (4.6)	0.013*

*p < 0.05

### Differences in cultural competence across White and non-White practitioners

The average total MCI score of all participants was *M* = 122.9 (*SD* = 14.0) (Table 2). The average scores for Subscale 1: Multicultural Counselling Skills, Subscale 2: Multicultural Awareness, Subscale 3: Multicultural Counselling Relationship, and Subscale 4: Multicultural Counselling Knowledge were *M* = 37.1 (*SD* = 5.0), *M* = 27.5 (*SD* = 5.0), *M* = 27.5 (*SD* = 5.0), and *M* = 33.7 (*SD* = 4.8), respectively. The total MCI score was higher in the non-White group compared to the White group, but the difference was not statistically significant (*M* = 125.5, *SD* = 14.6 vs. *M* = 121.5, *SD* = 13.6, *p* = 0.069). A similar trend was observed for Subscale 4: Multicultural Counselling Knowledge (*M* = 33.9, *SD* = 5.4 vs. *M* = 33.7, *SD* = 4.5, *p* = 0.433). While an inverse trend was observed for Subscale 1: Multicultural Counselling Skills (*M* = 36.7, *SD* = 5.0 vs. *M* = 37.3, *SD* = 5.0, *p* = 0.554), the differences between the non-White and White groups were not statically significant. However, the scores for Subscale 2: Multicultural Awareness (*M* = 29.5, *SD* = 5.0 vs. *M* = 26.4, *SD* = 4.6, *p* < 0.001) and Subscale 3: Multicultural Counselling Relationship (*M* = 25.4, *SD* = 3.5 vs. *M* = 24.2, *SD* = 3.3, *p* = 0.049) were significantly higher in the non-White group compared to the White group.

### Difference of Color-Blind Racial Attitudes Scale (CoBRAS) across White and non-White practitioners

The average total CoBRAS score of all participants was *M* = 43.8 (*SD* = 13.6) (Table 2). The scores for Factor 1: Unawareness of Racial Privilege, Factor 2: Unawareness of Institutional Discrimination, and Factor 3: Unawareness of Blatant Racism Issues were *M* = 18.8 (*SD* = 6.4), *M* = 14.4 (*SD* = 5.3), and *M* = 10.6 (*SD* = 0.3), respectively. The total CoBRAS score was significantly higher in the non-White group than the White group (*M* = 48.0, *SD* = 13.0 vs. *M* = 41.5, *SD* = 13.6, *p* = 0.007). A similar trend was observed for Factor 1: Unawareness of Racial Privilege (*M* = 20.9, *SD* = 6.4 vs. *M* = 17.6, *SD* = 6.2, *p* = 0.003) and Factor 3: Unawareness of Blatant Racism Issues (*M* = 11.9, *SD* = 4.6 vs. *M* = 10.0, *SD* = 4.0, *p* = 0.013). The score for Factor 2: Unawareness of Institutional Discrimination was also higher in the non-White group than the White group (*M* = 11.9, *SD* = 4.6 vs. *M* = 10.0, *SD* = 4.0, *p* = 0.059), however, the difference was not statistically significant. Similarly, no significant multicollinearity was observed in the model (Variance Inflation Factor -VIF for CoBRAS factor 1 = 1.00, CoBRAS factor 2 = 3.663 and CoBRAS factor 3 = 3.83).

### Predictors of cultural competence

Table 3 summarises the variables predicting participants’ cultural competence. Self-deceptive enhancement (SDE: Subscale 1 of BIDR), the age of participants, attendance of cultural competence-related workshops, conferences, or training sessions since the beginning of mental health care practice, and Unawareness of Blatant Racism Issues (UBRI: Factor 3 of CoBRAS) were observed to contribute significant variance to multicultural competence. The total proportion of variance for the dependent variable, which is explained by the independent variables in the model at Step 4, was calculated as total *R*^2^ = 0.374.

**Table 3:** Hierarchical multiple regression analysis predicting cultural competence

Variable	*B*	*SE B*	β	t	*R*^2^	∆R^2^	∆*F*	*df*
Step 1					0.141	0.141	10.706***	(2,130)
BIDR Subscale 1: Self Deceptive Enhancement (SDE)	1.378	0.433	0.306	3.183**				
BIDR Subscale 2: Impression Management (IM)	0.346	0.302	0.110	1.148				
Step 2					0.207	0.065	3.485*	(3,127)
Age	0.299	0.101	0.239	2.968**				
Ethnicity (being White)	1.890	2.934	0.066	0.644				
Participants speaks another language other than English	− 2.742	3.031	− 0.095	− 0.905				
Step 3					0.278	0.072	4.102**	(3,124)
Number of non-White clients per week	1.805	1.001	0.143	1.804				
Perception of preparedness to provide culturally competent services following formative training	− 0.695	1.006	− 0.054	− 0.691				
Number of cultural competence-related continuing professional development	3.350	1.301	0.221	2.574*				
Step 4					0.374	0.095	6.142***	(3,121)
CoBRAS Factor 1 (URP-Unawareness of Racial Privilege)	0.117	0.211	0.054	0.557				
CoBRAS Factor 2 (UID Unawareness of Institutional Discrimination)	0.413	0.253	0.158	1.629				
CoBRAS Factor 3 (UBRI Unawareness of Blatant Racism Issues)	− 1.323	0.329	− 0.410	− 4.023 ***				

*n* = 139, *p < 0.05. **p < 0.01. ***p < 0.001

Self-deceptive enhancement was observed to contribute to the variance of cultural competence, *F* (2,130) change = 10.706, *p* < 0.001, *R*^2^ = 0.141, and *R*^2^ change = 0.141, but not Impression Management. In Step 2, age was the only variable which contributed to the variation in cultural competence in addition to Self-Deceptive Enhancement from Step 1, *F* (3,127) change = 3.485, *p* = 0.018, *R*^2^ = 0.207, and *R*^2^ change = 0.065. The ethnicity of the participants (White and non-White) and the ability of the participants to speak a language other than English did not contribute to variance in cultural competence. Similarly, in Step 3, variables related to practitioners’ cultural competence experience were added to social desirability (BIDR subscales), age, ethnicity, ability to speak another language other than English. Cultural competence experience variables included: attendance at cultural competence-related workshops, conferences, or training sessions; number of non-White clients seen per week; and perceived preparedness to work with non-White clients following qualifying mental health training.

Step 3 yielded additional significant variance in cultural competence, *F* (3,124) change = 4.102, *p* = 0.008, *R*^2^ = 0.278, and *R*^2^ change = 0.072. However, the number of non-White clients per week and the belief that professional mental health training sufficiently prepared them to provide culturally competent services for non-White clients did not contribute any significant effect on cultural competence. In the final step (Step 4), the three subscales of CoBRAS were added in addition to all the variables in the previous steps. The analysis revealed that Unawareness of Blatant Racism Issues, together with the variables added in Steps 1 to 3, contributed to additional significant variance in cultural competence with a large effect size, F (3,121) change = 6.142, *p* < 0.001, R^2^ = 0.374, and R^2^ change = 0.095.

## Discussion

The purpose of this study was to identify the factors that influence Australian mental health practitioners’ cultural competence. The findings indicate that practitioners in this study valued cultural competence training given their high level of engagement in this type of continuing professional development. This may be because the majority engaged with at least one non-White client a week and/or wanted to maintain and/or improve their ability to provide culturally competent services to their non-White clients [44, 55].

Participants in the current study reported a similar level of cultural competence to those reported in previous studies. The MCI total score (*M* = 122.9, *SD* = 14.0) for the sample is approximately equivalent to data reported by Bellini [78] (*M* = 125.1, *SD* = 14.32) and Green et al. [79] (*M* = 126.71) for rehabilitation counselors (*n* = 372) and social workers (*n* = 344), respectively. The findings also indicate that non-White practitioners had higher levels of multi-cultural awareness and better multi-cultural counselling relationships with non-White people compared to White practitioners. This finding is consistent with research from other countries where non-White health practitioners demonstrate higher levels of cultural competence [32, 33]. Extant literature indicates that non-White people have personal experiences related to the various challenges that Whiteness presents in their own lives (e.g., being teased because of their race, ethnicity or religion as children) which may result in a high level of awareness of Whiteness and its impact on non-White people [47, 81–83]. Consequently, non-White people are more likely to build a more robust connection with non-White clients due to their increased ability to be empathetic to their clients’ experiences particularly with regards to discrimination-related distress [81, 84, 85]. Notably, comprehensive cultural competence training can moderate this discrepancy in cultural competency between White and non-White practitioners [32]. For example, Chao [32] found that following comprehensive cultural competence training, White practitioners’ cultural competence levels matched those of their non-White counterparts. Chao’s research demonstrated that training changes the association between race/ethnicity and cultural competence because such training increases mental health practitioners’ awareness of self and others and the impact of Whiteness on non-White people.

In line with international samples of mental health practitioners, the current sample demonstrated low to moderate levels of colour-blind racial and ethnic attitudes with average CoBRAS total scores of *M* = 43.8 (*SD* = 13.6). For instance, Neville et al. [31] reported that their sample of 152 American mental health workers and trainees “held low to moderate levels of color-blind racial beliefs” (p. 483) with mean CoBRAS score of *M* = 48.59 (*SD* = 12.79). Interestingly, non-White practitioners in the current study were more likely to be in denial about racial privilege and the pervasiveness of racial/ethnic issues in Australia compared to their White counterparts. This may be because the non-White practitioners in this study consider themselves, at least to some degree, beneficiaries of Whiteness. This may include their ability to access limited and competitive opportunities for higher education, their ability access health and social benefits enjoyed by those in higher socioeconomic brackets and the power and elevated social position that their career capital offers them [18, 19, 86]. Non-White practitioners working with a diversity of clients may therefore see parallels between White and non-White clients from low socioeconomic backgrounds and be less likely to attribute disadvantage to experiences of racial/ethnic discrimination [22]. Additionally, non-White practitioners may not want to buy-in to the idea that Australia suffers from pervasive and insurmountable institutional racism as this may hinder their own wellbeing and ability to encourage their non-White clients towards a fulfilling life.

In this study, the average BIDR Subscale 1: Self Deceptive Enhancement (SDE) score of all participants was *M* = 4.2 (*SD* = 3.1) and the average BIDR Subscale 2: Impression Management (IM) score was *M* = 6.2 (*SD* = 3.1). These findings are in line with normal ranges reported in other counsellor samples including Schomburg and Prieto [87] who reported trainee couples therapists’ BIDR SDE and IM mean scores as *M* = 5.3 (*SD* = 3.21) and *M* = 6.68 (*SD* = 4.05), respectively. The results of the hierarchical multiple regression indicate that highly favourable perceptions of self and protection of self-image (BIDR Subscale 1: Self Deceptive Enhancement SDE) was positively associated with practitioner cultural competence (MCI total score). As such, an increase in a respondent’s self-deceiving positive perception of their self-image, or at least an honest but overly positive perception of self, was found to be linked with greater multicultural competence. This may be because practitioners who perceive themselves more positively may have a higher sense of confidence in their abilities and therefore rate themselves as more culturally competent [44]. It could also be that participants protected their self-image due to the fear of being perceived as culturally incompetent, or even worse, being perceived as a bigot and/or racist. This fear may be so strong, that practitioners may be inclined to internalise the perception that they are more accepting, benevolent, self-aware and/or altruistic than they actually are to avoid facing the reality of their shortcomings. These interpretations align with other literature indicating that mental health care provider may experience a sense of identity threat when confronted with information and/or discussions about Whiteness, racism and privilege and their role in the perpetuation of prejudice and discrimination against non-White people [32, 88–90].

The relationship between social desirability and cultural competence was unexpected. While some researchers [41] proport that social desirability is an important issue in relation to self-report in cultural competence, others have not found any relationship between these variables [31, 32]. Following Chao (2013), the BIDR was included in this study. However, unlike Chao (2013) or Neville (2006) who found no relationship between these variables, the current study found a significant relationship between the self-deceptive enhancement subscale and higher MCI scores.

The hierarchical analysis also showed that participant’s age as well as the number of cultural competence-related workshops, conferences, or training sessions attended since the beginning of mental health care practice were positively associated with cultural competence. This finding suggests that cultural competence grows with clinical experience, regular engagement in training and maturity as a practitioner.

The hierarchical multiple regression also revealed that higher levels of unawareness of general and pervasive racial/ethnic discrimination (CoBRAS Factor 3: Unawareness of Blatant Racism Issues (UBRI)) are negatively associated with cultural competence (total MCI score). This finding mirrors those in other studies [30, 31, 77] and highlights that addressing race and racism is central to cultural competence training. Notably, the lack of training on race and racism may be why 48.9% of the sample were either neutral or disagreed that their qualifying training had prepared them to work with non-White people.

In summary, the study results demonstrate that non-White practitioners reported being more culturally aware and having better multi-cultural counselling relationships with non-White clients than their White counterparts. In this study, higher overall cultural competence is associated with practitioner’s age, greater attendance of cultural competence-related trainings and increased awareness of general and pervasive racial and/or ethnic discrimination. Practitioners with higher cultural competence are likely to perceive of themselves more favourably than those with lower levels of cultural competence.

### Implications and recommendations for training and practice

The findings of this study reinforce the need for continued engagement in cultural competence training across the full trajectory of a practitioner’s mental health career. The current study reiterates the importance of continuously addressing Whiteness by explicitly discussing White supremacy, privilege and power (for example) in Western mental health care settings and clearly identifying their impact on all people, not only those identifying as non-White [91–93]. This reflects a growing national focus on key components of cultural competence training and implementation, namely multicultural awareness, and relationships. For instance, the *Australian Heath Practitioner Regulation Agency* (AHPRA) encourages health practitioners not only to learn about why difference is a significant factor in therapeutic relationships but also how to engage with diverse clients in culturally competent ways [94]. While their current guidelines are focused on Indigenous Australians, many of the principles apply to other non-White groups. Given the lack of cultural competency guidelines from AHPRA relevant to the many other non-White groups in Australia, the development of such training and best-practice models is needed to ensure mental health practitioner’s preparedness to work with diverse populations. This recommendation is reinforced by the fact that cultural competence, is an integral part of all 15 health professions regulated by AHPRA and their professional codes of conduct.

Training should, however, be focused on addressing the particular needs of practitioners at various levels of experience and consider practitioner race and ethnicity. Pragmatically, practitioners could, for example, begin by completing demographic items as well as questions related to their existing counselling competence skills. Their responses would then determine allocation to training relevant to their age, racial/ethnic background as well as their professional experiences and expertise. For instance, younger, less experienced, White practitioners could engage in training that include frank discussions about Whiteness and anti-racism in mental health practice and services to increase their racial and cultural awareness. Additionally, those practitioners with higher levels of cultural competence can work on developing, implementing, or enhancing therapeutically safe mental health practises and those with lower levels can be introduced to the concepts and implications for their practice.

Aligned with the study findings, mental health care practices should actively engage with non-White populations to increase opportunities for cultural encounters between mental health practitioners and non-White clients [95, 96]. This can be done by offering home visits or providing services within community centres. Such engagement activities can also serve to decrease ethnic and racial colour-blindness amongst practitioners and offer opportunities for clients to develop effective counselling relationships with mental health care providers [89]. Given that many mental health practitioners, particularly psychologists, work in one-on-one settings engagement with their professional networks, case conferencing and clinical practice supervision can assist in increasing their awareness of general and pervasive racial and/or ethnic discrimination experienced by their clients [97–99]. Such forums would be especially effective where topics of race and Whiteness are directly addressed so that practitioners can be regularly challenged by their peers to reduce their racial and ethnic blindness and minimise the harm of practitioner ignorance on non-White clients.

### Limitations and future research

There are limitations to this study. First, the majority (76.7%) of the sample were psychologists which may limit the generalisability of the results to other mental health practitioners given that psychologists often work one-to-one with mental health clients. Further research including a more diverse sample of mental health practitioners would provide a more robust representation of cultural competence within the mental health sector. In addition, because of the limited number of non-White practitioners from diverse groups they were combined into a single group. Consequently, the results cannot be generalised to any particular group of non-White practitioners (e.g., Asian Australians, African Australians, Indigenous Australians). Additionally, the sample may be limited by selection bias with participants being specifically interested in the topic of cultural competence and/or those invested in the health outcomes of non-White clients. Research including a more diverse sample of mental health professionals may provide more information about the relationships between cultural competence and other variables like sex, age, duration of practice, and ethnicity. With a more diverse sample between groups difference can be explored more rigorously.

Second, the collected data drew on mental health practitioners’ self-report. It is well documented that self-report may not reflect actual levels of cultural competence [38, 40, 100]. Given that this study found a positive correlation between self-deceptive enhancement and cultural competence, additional methods for determining practitioner cultural competence are needed. This may include a comparison between clients’ perceptions of their therapist’s cultural competence versus their practitioners’ self-perceptions [101–103]. Further, observations and/or analysis of recordings of mental health sessions can be used to determine the provider’s cultural competence. While these recommendations present their own methodological limitations, they may be more in line with a culturally competent approach [104]. Self-report may also contribute to the surprising relationships found between socially desirable responding and cultural competence. As such, future studies with larger and more diverse samples should examine the association between social desirability and cultural competence.

## Conclusion

This is the first Australian study to explore the complex relationships among mental health care practitioners’ sociodemographic variables, cultural competence, multicultural training, colour-blind racial attitudes, and social desirability. Additionally, it is one the few studies to use a series of regressions to analyse diverse variables and their effect on cultural competence. The study sheds light on the attributes of mental health practitioners in Australia working with diverse clients while also navigating their own diversity and difference. The findings highlight that the current one-size-fits-all and skills-development approach to cultural competence training ignores the significant role that practitioner diversity and differences play in the counselling relationship. The recommendations from this study can inform clinical educators and supervisors about the importance of continuing professional development relevant to practitioners’ age, professional experience, and ethnic/racial background.

## Data Availability

Upon request, all relevant raw data will be freely available to any scientist wishing to use them for non-commercial purposes and without breaching participant confidentiality.

## Abbreviations

MCI: Multicultural Counselling Inventory
CoBRAS: Color-blind Racial Attitudes Scale
BIDR: Balanced Inventory of Desirable Responding
AHPRA: Australian Health Practitioner Regulation Agency

## Footnotes

1 The capitalisation of the ‘W’ in White and non-White is one of contentious scholarly debate. A capital W is used in this paper to acknowledge the racialisation and concomitant characterisation of ethnic, religious, cultural and linguistic affiliation in terms of proper nouns used to construct identities and not simply to inaccurately describe phenotypical traits (see also [3]).

2 It is acknowledged that other terms are increasingly being used in Australia and internationally to describe minority peoples. This includes terms like culturally and linguistically diverse (CALD) or Black, Indigenous and People of Colour (BIPOC). Importantly, the term CALD is widely criticised for its lack of specificity and erasure of various minority identifiers including ethnicity and religion. While the term BIPOC is more specific, again it is criticised for its lack of inclusion of religious diversity as well as its descriptive nature which sanitises and/or ignores the realities of power, supremacy and privilege within Whiteness. These terms also fail to identify White people and explicitly name Whiteness. This invisibility of Whiteness perpetuates its invincibility in mental health training, practice and research and negatively effects non-White (and White) people’s mental health outcomes (see [8, 9]).

3 It is acknowledged that these somewhat simplistic, skill-based and practitioner-focused constructions of cultural competence are limited in their recognition of the impact of racial dichotomies and hierarchies that result in the perpetuation of disadvantage and devaluation of non-White frameworks, perspectives or people [22].

4 Participants were asked to select any and all relevant ethnic identifiers including Asian, Pacific Islander, African, Latin American, Western European, Eastern European, Aboriginal and/or Torres Strait Islander, Indigenous Canadian and/or American, Middle Eastern, Mediterranean and Caucasian. Those who chose Caucasian and/or Western European were categorised as White for the purposes of analysis in line with our previous research in which participants of Western European descent self-identified as White.

5 To ensure conceptual clarity survey participants were presented with the definition of non-White people indicated in the Background section of this paper.
